# Mining the Prognostic Value of HNRNPAB and Its Function in Breast Carcinoma

**DOI:** 10.1155/2020/3750673

**Published:** 2020-05-13

**Authors:** Yun Cao, Wei Zhang, Yi-Ting Jin, Qiang Zou

**Affiliations:** Department of General Surgery, Huashan Hospital, Fudan University, Shanghai 200040, China

## Abstract

Heterogeneous nuclear ribonucleoproteins (HNRNPs) are crucial members in the pathogenesis and progression of numerous cancers. However, the expression pattern and clinical significance of HNRNPs in breast carcinoma (BC) remain to be investigated. In the present study, bioinformatic analysis identified HNRNPAB as the only commonly upregulated HNRNP in BC. Elevated expression of HNRNPAB was positively associated with more aggressive diseases and poorer survival rates in BC. Pathway analysis revealed that HNRNPAB coexpressed genes were enriched in the pathway of G2/M phase transition, and the expression level of HNRNPAB was strongly correlated with those of CCNB1, CDK1, CDC25A, and CDC25C. Experiments *in vitro* demonstrated that HNRNPAB knockdown suppressed cell proliferation and blocked the G2/M phase transition in BC. Taken together, this study provides the initial evidence that HNRNPAB may be employed as an innovative therapeutic target as well as a prognostic biomarker in BC patients.

## 1. Introduction

Breast carcinoma (BC) is the mostly diagnosed tumor and the major cause of cancer-associated mortality among women worldwide [1]. Despite the improved screening, diagnosis and treatment regimens, prognosis for patients with BC remains poor. Therefore, identification of more specific and sensitive biomarkers for early diagnosis and survival prediction, as well as novel therapeutic targets for effective therapy, is of great significance.

Heterogeneous nuclear ribonucleoproteins (HNRNPs) represent a large family of RNA-binding proteins and act as pivotal members in multiple aspects of RNA metabolism [2]. They assist in alternative splicing [3] and polyadenylation of precursor messenger RNA (mRNA) [4, 5], mRNA stability [6], mRNA nuclear export [7], and translational regulation [8–10]. Given their function diversity and complexity, HNRNPs have gained growing interest in disease research. The expressions of HNRNPs are altered in various cancers, suggesting their roles in oncogenesis. HNRNPC modulates the alternative cleavage and polyadenylation profiles in metastatic colon carcinoma [11]. HNRNPQ1 interacts with and enhances the translational efficiency of Aurora-A mRNA, thus contributing to cell proliferation in colorectal carcinoma [12]. HNRNPI regulates neonatal immune adaptation and prevents the development of colorectal carcinoma [13]. Previous studies have reported that HNRNPAB overexpression induces epithelial-mesenchymal transition and promotes the metastasis of hepatocellular carcinoma (HCC) via transcriptional regulation of SNAIL [14] and lncRNA-ELF209 [15]. HNRNPAB interacts with lncRNA-PCAT19 to activate a subset of cell cycle-related genes in the progression of prostate carcinoma [16]. However, the precise role of HNRNPAB in BC has been blurred.

Herein, a multitude of public datasets and platforms was utilized to determine the commonly upregulated HNRNPs in BC. HNRNPAB was identified as the only upregulated HNRNP in BC samples compared with noncancerous tissues. Higher expression of HNRNPAB indicated poorer survival in BC patients, and its association with clinicopathological characteristics was further analyzed using online databases. Pathway analysis of HNRNPAB coexpressed genes revealed that HNRNPAB might involve in cell cycle regulation, especially the G2/M phase transition. Moreover, HNRNPAB expression was strongly correlated with CCNB1, CDK1, CDC25A, and CDC25C expressions. Experiments *in vitro* confirmed that HNRNPAB knockdown could impede the proliferation capacity of BC cells and induce the G2/M phase arrest.

## 2. Materials and Methods

### 2.1. GEPIA Database Analysis

GEPIA (http://gepia.cancer-pku.cn/) is an interactive web server for analyzing the RNA sequencing expression data of 9736 tumors and 8587 normal samples from the TCGA and the GTEx projects, using a standard processing pipeline [17]. GEPIA was used to obtain upregulated genes in the TCGA-BRCA database via ANOVA. All overexpressed genes with significance met the criterion of combined *p* value < 1*E*-4 and log (fold change, FC) > 1. Spearman's correlations between CCNB1, CDK1, CDC25A, CDC25C, and HNRNPAB were downloaded from the website.

### 2.2. GEO

The GSE15852 microarray data was obtained from GEO (http://www.ncbi.nlm.nih.gov/geo/). The gene expression profile of 43 pairs of BC and normal tissue samples was determined. GEO2R was applied to identify all upregulated genes in BC tissues as opposed to normal breast tissues. All upregulated genes with significance met the criterion of combined *p* value < 0.05 and logFC > 1.

### 2.3. Oncomine Database Analysis

The Oncomine database (https://www.oncomine.org), an online platform that incorporates 715 independent datasets and 86733 samples [18], was utilized to evaluate the expression patterns of HNRNPAB in various tumor samples. The HNRNPAB mRNA level in BC samples was compared with that of their matched normal samples using 8 microarray datasets from 3 cohorts. The fold change of HNRNPAB expression was presented in box plots. The filters and thresholds used to obtain the datasets were set as follows: analysis type: cancer vs. normal analysis; *p* value: 1*E*-04; FC: 1.5; gene rank: 10%, data type: mRNA.

### 2.4. Breast Cancer Gene-Expression Miner v4.3 (bc-GenExMiner v4.3) Analysis

The bc-GenExMiner v4.3 (http://bcgenex.centregauducheau.fr/) is a statistical mining tool of published annotated breast cancer transcriptomic data and RNA-seq [19]. The statistical analyses are grouped in three modules: expression, prognosis, and correlation [20]. The bc-GenExMiner v4.3 correlation module was used to analyze the correlations between HNRNPAB mRNA expression and clinicopathological parameters in BC patients.

### 2.5. cBioPortal Database Analysis

The cBioPortal for Cancer Genomics (http://www.cbioportal.org/) is a web resource for exploring, visualizing, and analyzing multiple-dimensional cancer genomics data [21]. The Breast Invasive Carcinoma (TCGA, Provisional) study was selected, and a list of genes correlated with HNRNPAB was acquired from the website. The filters and thresholds used to obtain the gene list were given below: analysis type: mRNA expression (RNA Seq V2 RSEM vs. RNA Seq V2 RSEM); *p* value: 0.05; Spearman's correlation: 0.5.

### 2.6. PrognoScan Database Analysis

The PrognoScan database (http://www.prognoscan.org/) is a web-based platform that evaluates the relationship between candidate gene expression and prognosis in cancer patients [22]. Hazard ratios, 95% confidence intervals, and Cox *p* values were automatically calculated by the website.

### 2.7. Reactome Database Analysis

The Reactome website (http://reactome.ncpsb.org/) provides bioinformatic tools for pathway visualization and interpretation. The core unit of the Reactome data model is the reaction. Entities participating in the reactions form a network of biological interactions and are grouped into pathways [23]. Genes coexpressed with HNRNPAB were assessed using the Reactome Pathway Browser.

### 2.8. Cell Culture and Transfection

Human BC cell lines MCF7 and MDA-MB-231 were purchased from the Chinese Institute of Biochemistry and Cell Biology (China). MCF7 and MDA-MB-231 cells were maintained in Dulbecco's Modified Eagle's Medium and L-15 Medium (Gibco, USA) with 10% fetal bovine serum (FBS) (Gibco, USA) at 37°C with 5% CO_2_, respectively. Lentivirus vectors expressing short hairpin RNA, as well as the scramble controls, were purchased (GeneChem, China) and named as sh-HNRNPAB and sh-NC. The sequence of sh-HNRNPAB was as follows: 5′-CCGGTGAATTGCCAATGGATCCAAACTCGAGTTTGGATCCATTGGCAATTCATTTTTG-3′. Cells were transfected with lentivirus vectors for 48 hours and further selected using 2 *μ*g/mL puromycin (Selleck, USA). The efficiency of HNRNPAB knockdown was confirmed via western blot.

### 2.9. Western Blot Analysis

Total protein was harvested from cells using RIPA lysis buffer (Beyotime, China). Protein samples were separated by sodium dodecyl-sulfate polyacrylamide (SDS-PAGE) gel and transferred to polyvinylidene fluoride (PVDF) membranes. Blots were washed and incubated with antibodies. The protein bands were then visualized with an ECL detection system. The primary and secondary antibodies utilized in this study were listed as follows: anti-GAPDH (60004-1-Ig, Proteintech, USA), anti-HNRNPAB Abcam, USA), anti-CDK1 (ab133327, Abcam, USA), anti-Cyclin B1 (ab32053, Abcam, USA), anti-CDC25A (sc-7389, Santa Cruz, USA), anti-CDC25C (ab32444, Abcam, USA), anti-ER (ab75635, Abcam, USA), anti-HER2 (ab134182, Abcam, USA), HRP-conjugated goat anti-mouse IgG (SA00001-1, Proteintech, USA), and HRP-conjugated goat anti-rabbit IgG (SA00001-2, Proteintech, USA).

### 2.10. Cell Proliferation Assay

Cell growth was detected with cell count kit-8 (CCK8) following the manufacturer's instruction (Yeasen, China). For the CCK8 assay, 1 × 10^3^ cells were seeded per well in quintuplicate in 96 well plates. After 1, 2, 3, 4, and 5 days, cells were changed with 100 *μ*L fresh medium added 10 *μ*L CCK8 reagent and incubated for 2 hours at 37°C. Absorbance of each well was measured at 450 nm at indicated time points.

### 2.11. Flow Cytometry

Transfected cells were starved for 24 hours and then maintained in fresh medium with 10% FBS for 24 hours. Cells were harvested and fixed in ice-cold 75% ethanol overnight at 4°C. Then, cells were suspended in PI/RNase staining buffer (BD Biosciences, USA) for 15 minutes in the dark. Cell cycle distribution was assessed using a flow cytometer (BD Biosciences, USA).

### 2.12. Statistical Analysis

Each experiment was performed for three times. Two-tailed Student's *t*-test was employed for *in vitro* data. Data were presented as means ± standard deviation (SD) and analyzed with GraphPad Prism 7.0 software (La Jolla, USA). *p* value < 0.5 was considered astatistically significant.

## 3. Results

### 3.1. Identification of Upregulated HNRNPs in BC Tissues

**Figure 1:**
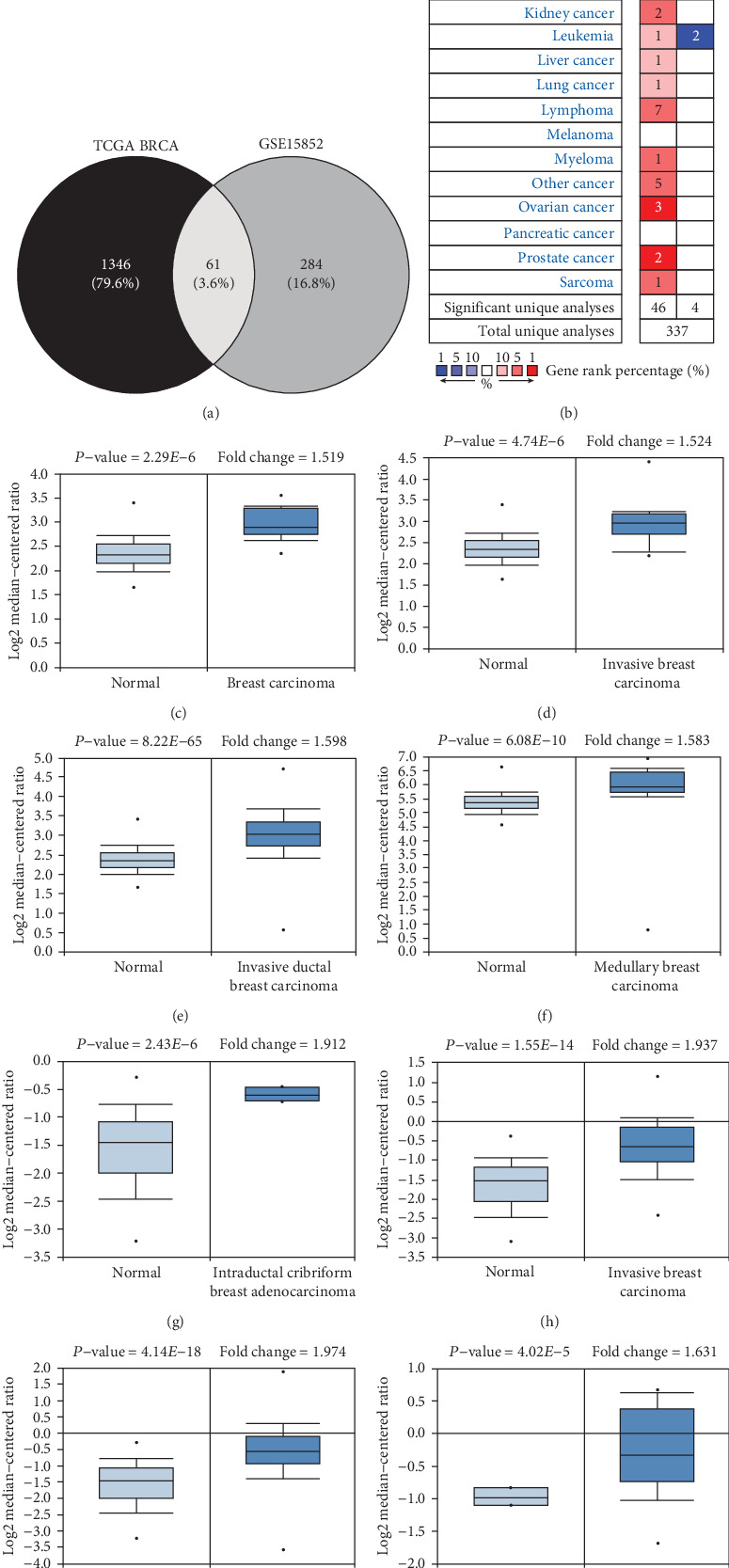
Upregulation of HNRNPAB in BC tissues. (a) Venn diagram of commonly upregulated HNRNPs in TCGA-BRCA and GSE15852. HNRNPAB was identified as the only overexpressed HNRNP in BC tissues compared to noncancerous tissues. (b) HNRNPAB was significantly upregulated in various malignancies including BC. (c-j) Box plots comparing the HNRNPAB mRNA level in normal tissues (left) and BC tissues (right) were generated from the Oncomine database. (k) HNRNPAB was amplified or upregulated in 6% of BC cases.

To identify overexpressed HNRNPs in BC, all upregulated genes in BC tissues versus noncancerous tissues were obtained from TCGA-BRCA database and GSE15852. 1406 overexpressed genes were found (logFC > 1.5, *p* < 0.0001) in TCGA-BRCA, while 344 upregulated genes were determined (logFC > 1.5, *p* < 0.05) in GSE15852. The Venn diagram revealed that HNRNPAB was the only HNRNP among the commonly upregulated genes in TCGA-BRCA and GSE15852 (see Figure 1(a)). Oncomine database analysis further confirmed that HNRNPAB was overexpressed in several solid tumors and hematological malignancies (see Figure 1(b)). In particular, HNRNPAB mRNA was statistically higher in BC tissues compared with that in matched normal tissues (see Figures 1(c)-1(j)). Details of HNRNPAB expression in these datasets are listed in Table 1. The cBioPortal database analysis demonstrated that HNRNPAB was amplified or upregulated in 6% of BC cases (see Figure 1(k)). Accordingly, the above lines of results suggested that HNRNPAB might act as an oncogene in the development of BC.

### 3.2. Clinical Significance of HNRNPAB in Patients with BC

**Figure 2:**
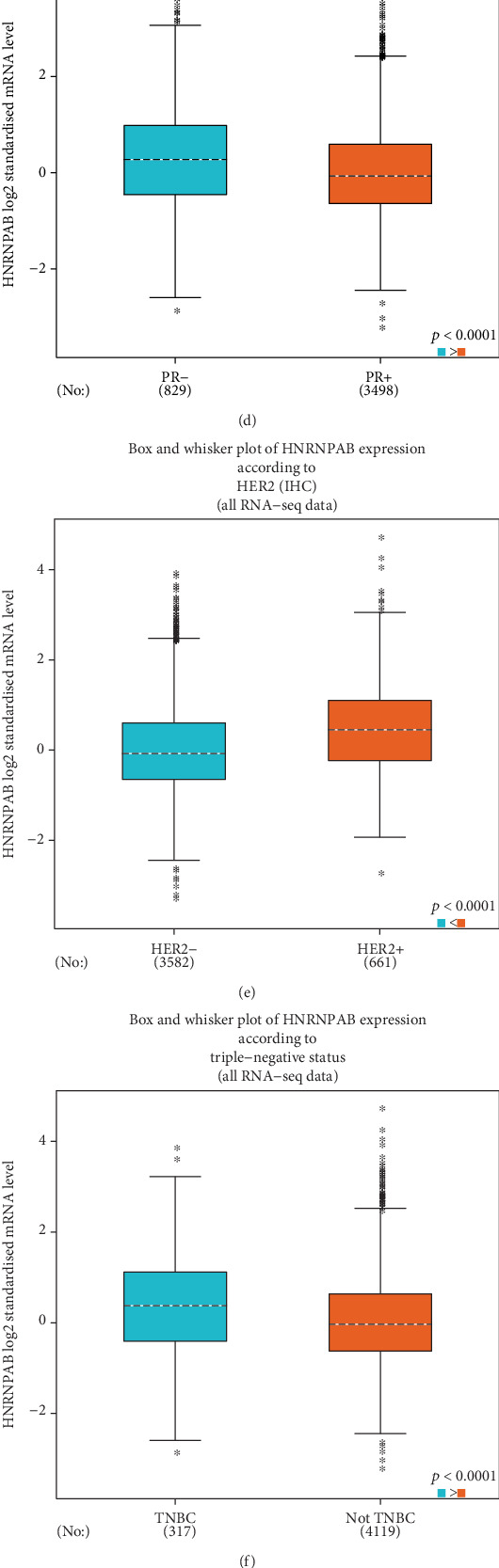
The correlations between HNRNPAB expression and clinicopathological parameters in BC patients were evaluated using bc-GenExMiner v4.3. Global significant difference between groups was determined using Welch's *t*-test to calculate *p* values, following with the Dunnett-Tukey-Kramer *t*-test.

To unveil the associations between HNRNPAB expression and patients' clinicopathological characteristics, data from bc-GenExMiner v4.3 was obtained and analyzed using Welch's *t*-test (see Table 2). With regards to age, HNRNPAB mRNA expression was higher in younger patients (*p* = 0.0029, see Figure 2(a)). Furthermore, BC patients with a positive nodal status exhibited higher HNRNPAB mRNA expression compared with those with negative nodal status (*p* < 0.0001, see Figure 2(b)). Estrogen (ER) status and progesterone (PR) status were negatively correlated with the HNRNPAB mRNA level (*p* < 0.0001, see Figures 2(c) and 2(d)). Conversely, HNRNPAB mRNA expression was prominently increased in patients with positive HER2 status (*p* < 0.0001, see Figure 2(e)). Patients with triple-negative BC (TNBC) exhibited higher HNRNPAB mRNA expression than non-TNBC patients (*p* < 0.0001, see Figure 2(f)). Our results also found that the HNRNPAB mRNA level was upregulated in patients with basal-like BC, comparing with those without basal-like BC (*p* < 0.0001, see Figure 2(g)). In terms of Scarff-Bloom-Richardson (SBR) grade and Nottingham prognostic index (NPI) grade, higher expression of HNRNPAB was significantly associated with more advanced SBR grade and NPI grade (*p* < 0.0001, see Figures 2(h) and 2(i)).

### 3.3. High HNRNPAB Expression Is Associated with Poor Prognosis in Patients with BC

**Figure 3:**
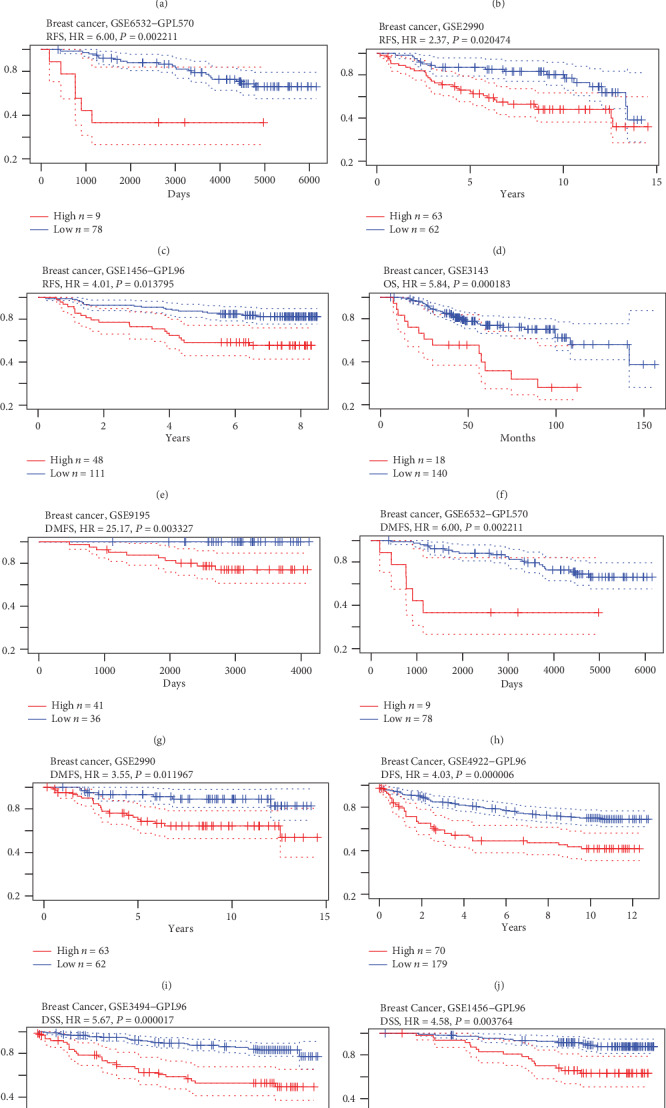
The prognostic role of HNRNPAB in BC patients. Survival curve analyses were performed with PrognoScan datasets. Hazard ratios, 95% confidence intervals, and Cox *p* values were automatically obtained from the website. Higher expression of HNRNPAB indicated unfavorable outcomes in BC patients.

The prognostic value of HNRNPAB in patients with BC was evaluated using the PrognoScan website. Survival curve analysis illustrated that the higher HNRNPAB level was remarkably correlated with shorter relapse-free survival (RFS), overall survival (OS), distant metastasis-free survival (DMFS), disease-free survival (DFS), and disease-specific survival (DSS) in BC, thus suggesting that HNRNPAB expression might be predictive of clinical outcomes in BC patients (see Figure 3 and Table 3).

### 3.4. The Potential Pathways of HNRNPAB in the Development of BC

**Figure 4:**
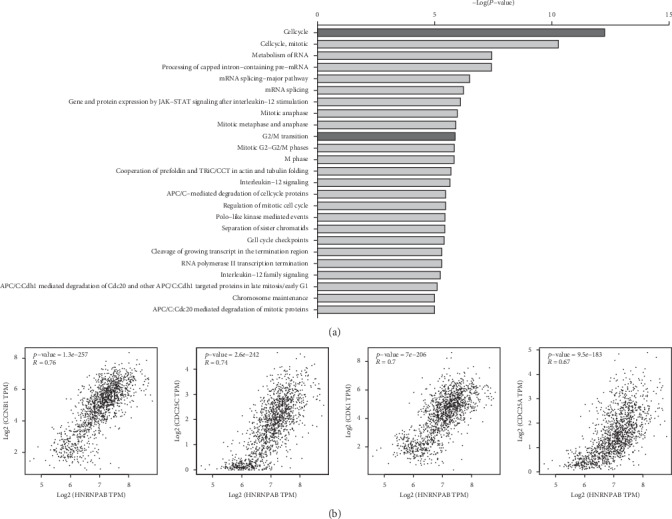
Pathway enrichment analysis of HNRNPAB coexpressed genes using Reactome platform. (a) HNRNPAB coexpressed genes were highly enriched in the pathways of cell cycle and G2/M phase transition. (b) The mRNA level of HNRNPAB was strongly correlated with those of CCNB1, CDK1, CDC25A, and CDC25C.

To clarify the biological functions and signaling pathways of HNRNPAB in tumorigenesis, pathway enrichment analysis of HNRNPAB-associated genes from TCGA-BRCA was performed using the Reactome database. The analysis identified the top 25 pathways in which HNRNPAB might be involved, including cell cycle and G2/M phase transition (see Figure 4(a) and Table 4). Furthermore, HNRNPAB mRNA expression was strongly correlated with CCNB1 (*R* = 0.76; *p* = 1.3E − 257), CDC25C (*R* = 0.74; *p* = 2.6E − 242), CDK1 (*R* = 0.7; *p* = 7E − 206), and CDC25A (*R* = 0.67; *p* = 9.5E − 183) mRNA expressions (see Figure 4(b)). Cyclin B1, cyclin-dependent kinase 1 (CDK1), cell division cycle 25A (CDC25A), and cell division cycle 25C (CDC25C) were G2/M-phase-related proteins, indicating that HNRNPAB might participate in the regulation of G2/M phase transition in BC.

### 3.5. HNRNPAB Exerts Promotive Effects on BC Cell Proliferation and Cell Cycle Progression

**Figure 5:**
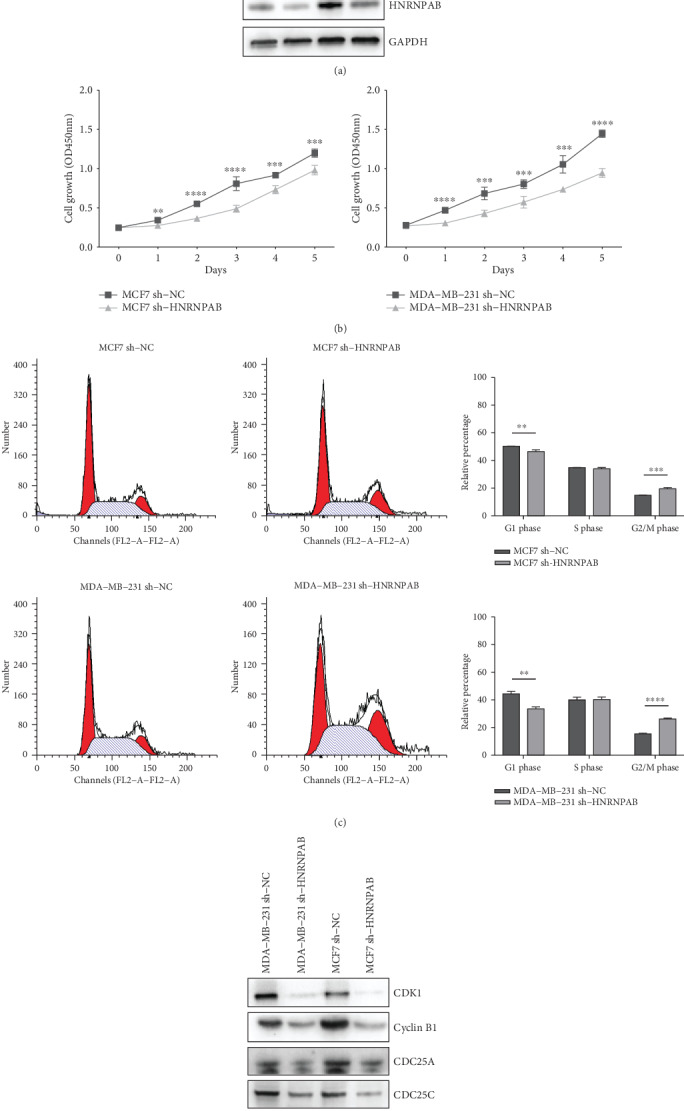
Experiments *in vitro* revealed the promotive effect of HNRNPAB on BC cells. (a) Western blot analysis of the efficiency of HNRNPAB knockdown. (b) HNRNPAB knockdown impeded the growth of BC cells. (c) HNRNPAB knockdown caused G2/M phase arrest in BC cells. (d) Western blot analysis of key regulatory molecules in G2/M phase transition in indicated BC cells. Data were shown as means ± SD, ^∗∗^*p* < 0.01; ^∗∗∗^*p* < 0.001; ^∗∗∗∗^*p* < 0.0001.

To determine the biological functions of HNRNPAB in BC progression, HNRNPAB was stably knockdown in MCF7 and MDA-MB-231 cells. The decreased expression of HNRNPAB was verified via western blot (see Figure 5(a)). The CCK8 assay demonstrated that HNRNPAB knockdown significantly suppressed the growth of BC cells (see Figure 5(b)). Flow cytometry was further conducted to investigate the effect of HNRNPAB on BC cell cycle distribution. Cell cycle analysis revealed that HNRNPAB knockdown led to an accumulation of cells in the G2/M phase, while the percentage of cells entering the G1 phase was reduced (see Figure 5(c)). Cyclin B1, CDK1, CDC25A, and CDC25C are key regulators indispensable for G2/M phase transition. CDK1/Cyclin B1 complex triggers mitosis via phosphorylation of multiple substrates, while CDC25A/C are protein phosphatases responsible for activation of CDK1. Western blot analysis confirmed that the protein levels of Cyclin B1, CDK1, CDC25A, and CDC25C were decreased in HNRNPAB-knockdown cells (see Figure 5(d)). These results were consistent with those of flow cytometry, indicating that HNRNPAB knockdown brought about the G2/M phase arrest in breast cancer cells.

## 4. Discussion

BC is the most common malignant tumor among women worldwide [1]. Despite the great achievements in screening, diagnosis and therapy for BC, novel therapeutic targets as well as predictive indicators are urgently required. The HNRNP family members are frequently altered in numerous malignancies and act as crucial players in cancer occurrence and progression [24]. However, the expression pattern and prognostic value of HNRNPs in BC have yet to be elucidated.

The present study screened out the commonly upregulated HNRNPs in TCGA-BRCA and GSE15852 using the Venn diagram. HNRNPAB was identified as the only overexpressed HNRNP in BC tissues relative to normal breast tissues. Zhou et al. reported that worse OS and a higher recurrence rate were observed in HCC patients with high HNRNPAB expression [14], suggesting that HNRNPAB served as a predictive indicator for HCC. Using the PrognoScan website, it was demonstrated that high HNRNPAB expression was significantly related with unfavorable RFS, OS, DMFS, DFS, and DSS in BC. Patient's age, ER status, and PR status were found to be negatively correlated to the HNRNPAB mRNA level. Conversely, nodal metastasis, HER2 status, TNBC and basal-like status, and SBR and NPI grades were positively associated with HNRNPAB expression. Collectively, these findings supported the role of HNRNPAB as a powerful predictor of poor patient outcomes in multiple malignancies including BC.

Given the aforementioned evidences that HNRNPAB is a potential oncogenic agent in BC, we therefore investigated the precise mechanisms of HNRNPAB in regulating malignant biological properties of BC cells. Pathway enrichment analysis of HNRNPAB coexpressed genes demonstrated that cell cycle was the top enriched pathway, following with RNA metabolism and the JAK-STAT signaling pathway, which were closely related with the occurrence and progression of BC.

The role of HNRNPAB in mediating BC cell aggressiveness was further evaluated *in vitro*. The CCK8 assay clearly revealed that downregulation of HNRNPAB could significantly repress the proliferation of BC cells. Hua et al. demonstrated that HNRNPAB interacted with the long isoform of lncRNA-PCAT19 and promoted cell growth in prostate carcinoma [16], which agrees with our data. We further performed flow cytometry to analyze the effect of HNRNPAB on cell cycle and revealed that HNRNPAB knockdown blocked the G2/M phase transition in BC cells. Western blot analysis confirmed that Cyclin B1, CDK1, CDC25A, and CDC25C were decreased in HNRNPAB-knockdown cells. These results were consistent with those of pathway analysis. Taken together, our observations implied that upregulation of HNRNPAB caused enhanced aggressiveness of BC cells; however, further studies on the underlying mechanisms are required.

## 5. Conclusions

This study was the first to illustrate that HNRNPAB was the only HNRNP commonly upregulated in BC tissues compared with adjacent normal tissues. High HNRNPAB expression was associated with unfavorable clinical outcomes in patients with BC. The HNRNPAB expression level was negatively correlated with patient's age, ER status, and PR status and positively associated with nodal, HER2, TNBC, and basal-like status. Additionally, HNRNPAB expression was increased in patients with more advanced SBR and NPI grades. Furthermore, bioinformatic analysis revealed that HNRNPAB might be involved in G2/M phase transition and was strongly associated with CCNB1, CDK1, CDC25A, and CDC25C. Experiments *in vitro* demonstrated that HNRNPAB knockdown suppressed the cell proliferation capacity and impeded the G2/M phase transition. To summarize, this study sheds new light on better understanding the fundamental role of HNRNPs in BC progression and discovers HNRNPAB as an innovative therapeutic target and prognostic biomarker for BC patients.

## Figures and Tables

**Table 1:** Oncomine analysis of HNRNPAB expression in BC.

No.	Cohort name	Data type	Sample (n)	Fold change	*p* value
1	Curtis Breast Statistics	mRNA	Breast carcinoma (14) vs. normal (144)	1.519	2.29*E*-06
		mRNA	Invasive breast carcinoma (21) vs. normal (144)	1.524	4.74*E*-06
		mRNA	Invasive ductal breast carcinoma (1556) vs. normal (144)	1.598	8.22*E*-65
		mRNA	Medullary breast carcinoma (32) vs. normal (144)	1.583	6.08*E*-10

2	TCGA Breast Statistics	mRNA	Intraductal cribriform breast adenocarcinoma (3) vs. normal (61)	1.912	2.43*E*-06
		mRNA	Invasive breast carcinoma (76) vs. normal (61)	1.937	1.55*E*-14
		mRNA	Invasive ductal breast carcinoma (389) vs. normal (61)	1.974	4.14*E*-18

3	Zhao Breast Statistics	mRNA	Invasive ductal breast carcinoma (35) vs. normal (3)	1.631	4.02*E*-05

**Table 2:** The associations between HNRNPAB mRNA expression and clinicopathological parameters in BC.

Variable	Case	mRNA expression	*p* value
Age (years)			0.0029
≤51	1099	↑	
>51	3209	—	
Nodal status			<0.0001
Positive	1646	↑	
Negative	2416	—	
ER (IHC)			<0.0001
Positive	3911	—	
Negative	552	↑	
PR (IHC)			<0.0001
Positive	3498	—	
Negative	829	↑	
HER2 (IHC)			<0.0001
Positive	661	↑	
Negative	3582	—	
Triple-negative status			<0.0001
Not TNBC	4119	—	
TNBC	317	↑	
Basal-like status			<0.0001
Not basal-like	3837	—	
Basal-like	832	↑	
SBR grade			<0.0001
SBR1	544	—	
SBR2	1699	↑	
SBR3	1374	↑	
NPI grade			<0.0001
NPI1	1173	—	
NPI2	1525	↑	
NPI3	416	↑	

Note: ER: estrogen receptor; PR: progesterone receptor; HER2: human epidermal growth factor receptor 2; IHC: immunochemistry; TNBC: triple-negative breast carcinoma; SBR: Scarf-Bloom-Richardson; NPI: Nottingham prognostic index.

**Table 3:** The expression of HNRNPAB and survival rates in BC patients.

Dataset	Endpoint	Probe ID	Sample number	Cox *p* value	HR	95% CI
GSE4922-GPL96	Disease free survival	201277_s_at	249	0.000006	4.03	2.21-7.36
GSE3494-GPL96	Disease specific survival	201277_s_at	236	0.000017	5.67	2.57-12.51
GSE3143	Overall survival	38094_at	158	0.000183	5.84	2.32-14.72
GSE1456-GPL96	Relapse free survival	201277_s_at	159	0.001363	4.01	1.71-9.39
GSE12276	Relapse free survival	201277_s_at	204	0.001956	2.16	1.33-3.51
GSE6532-GPL570	Relapse free survival	201277_s_at	87	0.002211	6.00	1.90-18.89
GSE6532-GPL570	Distant metastasis free survival	201277_s_at	87	0.002211	6.00	1.90-18.89
GSE9195	Distant metastasis free survival	201277_s_at	77	0.003327	25.17	2.92-216.77
GSE1456-GPL96	Disease specific survival	201277_s_at	159	0.003764	4.58	1.64-12.81
GSE2990	Distant metastasis free survival	201277_s_at	125	0.011967	3.55	1.32-9.53
GSE9195	Relapse free survival	201277_s_at	77	0.013795	9.13	1.57-53.10
GSE2990	Relapse free survival	201277_s_at	125	0.020474	2.37	1.14-4.93

Note: HR: hazard ratio; CI: confidence interval.

**Table 4:** Pathway analysis of HNRNPAB-associated genes in Reactome database.

No.	Top 25 pathways in Reactome database	No. of input genes	*p* value	-log (*p* value)	FDR
1	Cell cycle	31/682	5.59E-13	12.25258819	3.03E-10
2	Cell cycle, mitotic	26/570	5.22*E*-11	10.2823295	1.41*E*-08
3	Metabolism of RNA	26/782	3.66*E*-08	7.436518915	5.05*E*-06
4	Processing of capped intron-containing pre-mRNA	15/256	3.74*E*-08	7.427128398	5.05*E*-06
5	mRNA splicing-major pathway	12/185	3.20*E*-07	6.494850022	3.46*E*-05
6	mRNA splicing	12/196	5.85*E*-07	6.232844134	5.27*E*-05
7	Gene and protein expression by JAK-STAT signaling after interleukin-12 stimulation	8/74	7.94*E*-07	6.100179498	6.11*E*-05
8	Mitotic anaphase	12/208	1.08*E*-06	5.966576245	6.61*E*-05
9	Mitotic metaphase and anaphase	12/211	1.25*E*-06	5.903089987	6.61*E*-05
10	G2/M transition	12/212	1.32*E*-06	5.879426069	6.61*E*-05
11	Mitotic G2-G2/M phases	12/214	1.45*E*-06	5.838631998	6.61*E*-05
12	M phase	16/390	1.47*E*-06	5.832682665	6.61*E*-05
13	Cooperation of prefolding and TRiC/CCT in actin and tubulin folding	6/37	2.01*E*-06	5.696803943	8.24*E*-05
14	Interleukin-12 signaling	8/85	2.20*E*-06	5.657577319	8.36*E*-05
15	APC/C-mediated degradation of cell cycle proteins	8/90	3.34*E*-06	5.476253533	1.09*E*-04
16	Regulation of mitotic cell cycle	8/90	3.34*E*-06	5.476253533	1.09*E*-04
17	Polo-like kinase-mediated events	5/23	3.62*E*-06	5.441291429	1.09*E*-04
18	Separation of sister chromatids	11/194	3.64*E*-06	5.438898616	1.09*E*-04
19	Cell cycle checkpoints	13/279	3.91*E*-06	5.407823243	1.09*E*-04
20	Cleavage of growing transcript in the termination region	7/67	4.93*E*-06	5.307153081	1.23*E*-04
21	RNA polymerase II transcription termination	7/67	4.93*E*-06	5.307153081	1.23*E*-04
22	Interleukin-12 family signaling	8/97	5.74*E*-06	5.241088108	1.38*E*-04
23	APC/C:Cdh1-mediated degradation of Cdc20 and other APC/C:Cdh1 targeted proteins in late mitosis/early G1	7/72	7.84*E*-06	5.105683937	1.80*E*-04
24	Chromosome maintenance	8/105	1.01*E*-05	4.995678626	2.14*E*-04
25	APC/C:Cdc20-mediated degradation of mitotic proteins	7/75	1.02*E*-05	4.991399828	2.14*E*-04

## Data Availability

The datasets used and/or analyzed in the current study are available from the corresponding author upon reasonable request.
